# Association of Abnormal Renal Profiles with Subretinal Fluid in Diabetic Macular Edema

**DOI:** 10.1155/2022/5581679

**Published:** 2022-12-13

**Authors:** Xiaoyan Zhang, Xiaofeng Hao, Linan Wang, Like Xie

**Affiliations:** Department of Ophthalmology, Eye Hospital, China Academy of Chinese Medical Sciences, Beijing, China

## Abstract

**Purpose:**

To investigate the risk factors for subretinal fluid (SRF) in diabetic macular edema (DME).

**Methods:**

We reviewed the records of 66 patients with DME. Systemic parameters, including hypertension, glycosylated hemoglobin, serum fasting glucose, total cholesterol, triglycerides, and diabetic kidney disease, were evaluated. Renal parameters for diabetic kidney disease included serum albumin, serum creatinine, albuminuria, and estimated glomerular filtration rate. Ocular factors included visual acuity and diabetic retinopathy, and the following parameters are evaluated through optical coherence tomography examination: disorganization of the retinal inner layers, loss of ellipsoid zone, central subretinal fluid thickness, central macular thickness, and presence of SRF.

**Results:**

Higher albuminuria (odds ratio, 3.431; 95% confidence interval, 1.039–11.334; *P*=0.043) was associated with the presence of SRF in patients with DME. Lower serum albumin levels (beta = −14.028, se = 6.646, *P*=0.044) were associated with increased SRF thickness.

**Conclusions:**

Poor kidney function was associated with the presence of SRF in DME. Screening for SRF in DME in patients with higher albuminuria and lower serum albumin levels should be routinely performed.

## 1. Introduction

Diabetic macular edema (DME) is a major cause of visual impairment in patients with diabetes [1] and can be classified into cystoid, diffuse, serous, or mixed types.

DME occurs because of the disruption of the blood-retinal barrier. Diabetic kidney disease and DME may share a similar microvascular pathophysiology [2]. Serum albumin, creatinine, albuminuria, and estimated glomerular filtration rate (eGFR) have been used as markers of diabetic kidney disease. Previous studies have shown that albuminuria and macroalbuminuria are closely associated with DME [2–4]. However, eGFR does not seem to have any effect on the severity or pattern of DME [5, 6].

Subretinal fluid (SRF) is found in 15%–30% of DME cases [2]. Persistent SRF may be detrimental to the retinal pigment epithelium and photoreceptors [7]. Few studies have reported the association between SRF in DME and diabetic kidney disease. Koo et al. [8] found a significantly higher frequency of SRF in DME in patients with albuminuria. In this study, we investigated the association between diabetic kidney disease and the presence and severity of SRF in patients with diabetes.

## 2. Materials and Methods

This study adhered to the tenets of the Declaration of Helsinki. Ethical approval for this study was waived by the Local Ethics Committee of Eye Hospital, China Academy of Chinese Medical Sciences in view of the retrospective nature of the study.

### 2.1. Data Collection

Eyes that had undergone laser photocoagulation, intravitreal injection, or intraocular surgery within 3 months prior to the study were excluded. Patients with macular edema of other causes, such as retinal vein occlusion and uveitis, were also excluded. Finally, a total of 66 patients with DME who visited our department between September 01, 2017, and September 30, 2020, were retrospectively enrolled.

Patient characteristics, including age, gender, duration of diabetes, hypertension, visual acuity, serum fasting glucose, glycosylated hemoglobin (HbA_1c_), triglycerides, total cholesterol, serum albumin, serum creatinine, urinary albumin creatinine ratio, eGFR, and diabetic retinopathy (DR) severity, were recorded. Disorganization of the retinal inner layers, loss of ellipsoid zone, central SRF thickness, central macular thickness (CMT), and presence of SRF were documented using optical coherence tomography. Disorganization of the retinal inner layers was defined as the inability to distinguish the boundaries between the ganglion cell—inner plexiform layer complex, inner nuclear layer, and outer plexiform layer [9]. DR severity was categorized as mild nonproliferative retinopathy (mild NPDR), moderate NPDR, severe NPDR, and proliferative diabetic retinopathy. Overnight first-void urine samples were collected. Albuminuria was defined as a urinary albumin-to-creatinine ratio >30 mg/g. eGFR was calculated by using the chronic kidney disease epidemiology collaboration equation and was categorized into the following five groups: <30 mL/min/1.73 m^2^ (stage 1), 30–44 mL/min/1.73 m^2^ (stage 2), 45–59 mL/min/1.73 m^2^ (stage 3), 60–89 mL/min/1.73 m^2^ (stage 4), or >90 mL/min/1.73 m^2^ (stage 5). SRF, a categorical variable, was divided into two categories: the presence and absence of SRF. Hypertension was analyzed as a categorical variable, with patients divided into two categories according to the presence or absence of hypertension. Data from the worst eye were used for analyses.

### 2.2. Statistical Analyses

Statistical analyses were performed using SPSS Statistics for Windows, version 26.0 (IBM, Armonk, NY). Summary statistics included the mean ± standard deviation, where appropriate. Binary and multiple logistic regression models were used to assess the risk factors for SRF. Univariate and multivariable linear regressions were used to evaluate the correlation between central SRF thickness and clinical parameters. Univariate linear regression was used to evaluate the correlation between CMT and clinical parameters. *P* values <0.05 were considered statistically significant.

## 3. Results

A total of 66 patients with DME were enrolled in this study. The baseline characteristics of the patients are shown in Table 1. None of the patients had undergone dialysis. Of the total patients, 39 (59%) were women. The mean age was 58.2 ± 11.2 years, the mean duration of diabetes was 10.6 ± 6.9 years, the mean HbA_1c_ level was 8.0 ± 1.7%, and the mean CMT was 523.2 ± 154 *μ*m. SRF in DME was observed in 30 patients.

Table 2 shows the factors associated with the presence of SRF. Binary logistic regression analysis revealed that lower HbA_1c_ levels (odds ratio [OR], 0.727; 95% confidence interval [CI], 0.529–0.999; *P*=0.049) and higher albuminuria (OR, 3.571; 95% CI, 1.112–11.468; *P*=0.032) were associated with the presence of SRF among the 66 patients with DME. Multiple regression analysis revealed that only albuminuria was significantly associated (OR, 3.431; 95% CI, 1.039–11.334; *P*=0.043) with the presence of SRF. Visual acuity, DR severity, hypertension, serum fasting glucose, triglycerides, total cholesterol, eGFR, serum albumin, and serum creatinine showed no significant association with the presence of SRF.  Table 3 shows the factors associated with SRF thickness according to the univariate linear regression among the 30 patients with SRF. Lower serum albumin levels (beta = −14.028, se = 6.646, *P*=0.044) were associated with a greater SRF thickness.

Table 4 shows the factors associated with CMT. Univariate linear regression revealed that HbA_1c_ (beta = −42.17, se = 10.34, *P*=0.0001) and serum fasting glucose (beta = −13.24, se = 5.55, *P*=0.020) were associated with CMT among the 66 patients with DME. Multivariable linear regression revealed that a lower HbA_1c_ was significantly associated with a higher CMT (beta = −42.06, se = 13.28, *P*=0.002). However, albuminuria, eGFR, serum albumin, and serum creatinine were not associated with CMT.

## 4. Discussion

Our study results suggest that diabetic kidney disease plays an important role in the occurrence of SRF in DME. Higher albuminuria showed a better association with the presence of SRF than lower HbA_1c_ levels, although both were important risk factors for the presence of SRF.

Our data suggested that the presence of SRF had a good correlation with higher albuminuria, while serum albumin was not significantly different between the patients with or without SRF. In addition, we found that the severity of SRF was negatively correlated with serum albumin levels. Low serum albumin levels were associated with increased SRF thickness. Tsai et al. [7] reported that the presence of SRF was correlated with low serum albumin levels. This may be because the correlation of baseline SRF with serum albumin levels was performed to adjust for age and DR severity. Koo et al. [8] also reported a significantly higher frequency of SRF in DME in patients with albuminuria. Vascular hyperpermeability is a possible shared pathogenetic mechanism between the kidney (albuminuria) and the eye (SRF of DME). Fluid movement is governed by changes in pressure gradients. Advanced proteinuria with marked protein loss may result in lower intravascular osmotic pressure and higher hydrostatic pressure, leading to fluid retention in the subretinal space. In the early stages, serum albumin deficits may be compensated for by the increased production of albumin molecules in the liver, which may prevent hypoalbuminemia [10]. In the late stage, serum albumin deficits that could not be compensated for may be associated with increased SRF thickness. Additionally, according to our results, no associations could be found between albuminuria and CMT, which is similar to the findings of previous studies [11–13]. We inferred that patients with albuminuria may be prone to more fluid leakage from choroidal vessels and damaged retinal pigment epithelium, which eventually results in SRF in DME. Moreover, previously reported case series on SRF secondary to nephrotic syndrome demonstrated that SRF resolved following systemic furosemide treatment [14–16].

Contrary to the general belief [17], we observed that lower HbA_1c_ levels were more likely to result in SRF and were associated with a higher CMT. Data from a more recent study also indicated that lower HbA_1c_ levels are more likely to result in SRF in DME [11]. Other supportive evidence showed that better control of HbA_1c_ did not lead to a greater reduction in CMT in DME [18]. Furthermore, Falavarjani et al. [19] observed reduced CMT after meals compared with that before meals. There are several possible explanations for this finding. First, it may be because of the early worsening effect, which can be caused by the rapid reduction in HbA_1c_ levels [11]. Second, lowering serum glucose levels can lower the intravascular osmotic pressure. We inferred that the pressure gradient may have increased the tendency of the fluid to move from the intravascular to the retinal parenchyma and subretinal space. Thus, tight control of HbA_1c_ may be associated with higher CMT and the presence of SRF. However, further research with a larger sample size is needed to clarify this.

In the present study, no associations were found between eGFR and the severity spectrum of DME or the presence of SRF. Previous studies have also shown that eGFR does not seem to have any effect on the severity or pattern of DME [5, 6]. However, Man et al. [2] and Temkar et al. [5] found that lower eGFR was not associated with the presence or severity of DME. Additionally, we did not find any significant correlation between DR severity and the presence of SRF. This may be because retinopathy and nephropathy may have a similar pathology, and eGFR and DR may fluctuate over time. However, further research is required to clarify this.

Our study has some limitations. It was a retrospective study with a small sample size.

## 5. Conclusions

According to our study results, higher albuminuria was associated with the presence of SRF in patients with DME. Lower serum albumin levels were associated with greater SRF thickness. Hence, screening for SRF in DME in all patients with diabetic kidney disease should be emphasized.

## Figures and Tables

**Table 1:** Baseline clinical parameters of diabetic macular edema.

Parameters	Diabetic macular edema (*n* = 66)
Age (years)	58.2 ± 11.2 (32–86)
Female (no)	39
Diabetes duration (years)	10.6 ± 6.9 (0.25–30)
Hypertension (patients)	36
Fasting glucose level (mmol/L)	8.49 ± 3.32 (3.35–21.05)
HbA_1c_ (%)	8.0 ± 1.7 (5.4–11.7)
Triglyceride (mmol/L)	1.9 ± 1.8 (0.71–14.6)
Total cholesterol (mmol/L)	4.7 ± 1.3 (2.34–7.98)
Serum albumin (g/L)	41.0 ± 3.7 (32.7–48.3)
Serum creatinine (mg/dL)	1.3 ± 1.3 (0.5–10.69)
eGFR (ml/min/1.73 m^2^)	64.8 ± 24.6 (4–122)
Urinary albumin creatinine ratio (mg/g)	194.1 ± 223.7 (2.4–1201.5)
Disorganization of the retinal inner layers (patients)	24
Loss of the ellipsoid zone (patients)	6
CMT (*μ*m)	523.2 ± 154 (155–843)
Subretinal fluid (patients)	30
Diabetic retinopathy severity	
Mild NPDR	0
Moderate NPDR	16
Severe NPDR	25
PDR	25

HbA_1c_: glycated hemoglobin; eGFR: estimated glomerular filtration rate; CMT: central macular thickness; NPDR: non-proliferative diabetic retinopathy; PDR: proliferative diabetic retinopathy.

**Table 2:** Correlating factors for presence of subretinal fluid in patients with diabetic macular edema.

Parameters	Binary logistic regression analysis			Multiple logistic regression analysis		
	OR	95% CI	*P* value	OR	95% CI	*P* value
Age (years)	0.970	0.927–1.014	0.181	—	—	—
Diabetes duration (years)	0.964	0.896–1.038	0.33	—	—	—
Hypertension	1.5	0.563–3.997	0.417	—	—	—
Visual acuity	2.62	0.853–8.05	0.093	—	—	—
Fasting glucose level (mmol/L)	0.889	0.751–1.051	0.169	—	—	—
HbA_1c_ (%)	0.727	0.529–0.999	0.049	0.730	0.530–1.006	0.055
Triglyceride (mmol/L)	1.452	0.849–2.481	0.173	—	—	—
Total cholesterol (mmol/L)	0.897	0.621–1.295	0.561	—	—	—
Serum albumin (g/L)	0.951	0.831–1.089	0.469	—	—	—
Serum creatinine (mg/dL)	2.023	0.731–5.598	0.175	—	—	—
Albuminuria	3.571	1.112–11.468	0.032	3.431	1.039–11.334	0.043
eGFR	1.336	0.877–2.035	0.177	—	—	—
DR severity	0.991	0.531–1.849	0.977	—	—	—

**Table 3:** Univariate linear regression for correlating factors of subretinal fluid thickness.

Parameters	Beta	se	*P* value
Age (years)	1.299	2.487	0.606
Diabetes duration (years)	−1.157	5.770	0.843
Fasting glucose level (mmol/L)	−11.242	12.289	0.368
HbA_1c_ (%)	−30.529	16.591	0.077
Serum creatinine (mg/dL)	−1.07	15.695	0.946
Serum albumin (g/L)	−14.028	6.646	0.044
Total cholesterol (mmol/L)	−35.112	19.773	0.087
Triglyceride (mmol/L)	−1.833	11.203	0.871
Albuminuria	53.360	75.140	0.483
Hypertension	−48.972	56.926	0.397
eGFR	14.962	20.895	0.480
Visual acuity	81.498	58.892	0.177
DR severity	11.543	39.289	0.771

**Table 4:** Correlating factors for CMT in patients with diabetic macular edema.

Parameters	Univariate linear regression			Multivariable linear regression		
	Beta	se	*P* value	Beta	se	*P* value
Age (years)	−1.315	1.706	0.444	—	—	—
Diabetes duration (years)	−3.585	2.753	0.198	—	—	—
Fasting glucose level (mmol/L)	−13.24	5.55	0.020	−0.09	6.69	0.989
HbA_1c_ (%)	−42.17	10.34	0.0001	−42.06	13.28	0.002
Serum creatinine (mg/dL)	15.372	14.944	0.308	—	—	—
Serum albumin (g/L)	−1.508	5.332	0.778	—	—	—
Total cholesterol (mmol/L)	−25.39	13.93	0.073	—	—	—
Triglyceride (mmol/L)	2.4	10.48	0.820	—	—	—
Albuminuria	14.846	41.528	0.722	—	—	—
Hypertension	−0.25	38.366	0.995	—	—	—
eGFR	6.523	16.118	0.687	—	—	—
DR severity	12.426	24.560	0.615	—	—	—

## Data Availability

The data used to support the findings of this study are available from the corresponding author upon request.
